# Artificial intelligence as a digital analytic third: the SADAR framework for reflective supervision in psychotherapy

**DOI:** 10.3389/fpsyg.2026.1690291

**Published:** 2026-05-08

**Authors:** Sabrina Signorini, Walter Paganin

**Affiliations:** 1 Studio Psicologia Signorini, Guidonia Montecelio, Italy; 2 Università di Roma Tor Vergata, Rome, Italy

**Keywords:** digital analytic third, artificial intelligence, countertransference, internal multiplicity, psychodynamic AI, psychotherapy, reflective supervision, SADAR

## Abstract

The SADAR (Sistema Autoesplorativo Dialogogico Autentico Relazionale—Authentic Relational Dialogogic Self-Exploratory System) positions AI as a Digital Analytic Third for therapists’ post-session reflection and clinical supervision, rather than for direct patient interventions or automated clinical decision-making. The SADAR frames AI as a dialogogic symbolic co-presence that expands the therapist’s reflective space and deepens countertransferential awareness, without replacing professional judgment or human supervision. This perspective article defines the term “dialogogic,” presents a three-step reflective process (positioning, dialogogic prompting, and critical integration), and illustrates the framework’s potential and limitations through two clinical vignettes. The operational implementation is detailed in a companion brief report. All outputs must adhere to reflective supervision protocols and should never replace direct patient data.

## Introduction

In recent years, digital mental health has emphasized scalable interventions and process monitoring, primarily assigning AI a predominantly instrumental role (Fitzpatrick et al., 2017; Inkster et al., 2018). In parallel, there is growing international interest in frameworks that situate AI within psychodynamic thought, including discussions of the Artificial/Analytic Third and exploratory uses of large language models (LLMs) in psychodynamic formulation, as well as responsible evaluation in behavioral health (Rabeyron, 2025; Haber et al., 2024; Hwang et al., 2024; Kim et al., 2025; Stade et al., 2024). In this dialogue, the present article proposes a non-prescriptive conceptual framework that integrates the Analytic Third (Ogden, 1994), intersubjectivity (Benjamin, 2004), the transitional object (Winnicott, 1971), and reflections on the digital and subjectivity (Han, 2015; Esposito, 2011; Freud, 1955) to situate AI as a reflective, symbolic co-presence within the therapist’s reflective work.

While existing contributions have advanced the positioning of AI within psychodynamic thought, the SADAR occupies a distinct theoretical niche that has not been adequately addressed by prior frameworks. Haber et al. (2024) and Rabeyron (2025) conceptualized the Artificial Third primarily in relation to the patient-facing therapeutic dyad, focusing on how AI reshapes relational dynamics and clinical interactions. Hwang et al. (2024) and Kim et al. (2025) explored AI as a tool for psychodynamic formulation, evaluating AI-generated diagnostic outputs as clinical decision support. The SADAR differs from these frameworks across three dimensions: (a) scope—it is exclusively oriented toward the therapist’s internal reflection and clinical supervision, never extending to direct patient interaction or automated decision-making; (b) theoretical framing—AI is engaged as a reflective, symbolic co-presence that perturbs and amplifies the therapist’s reflective field, rather than generating outputs to be applied clinically; and (c) process structure—it operationalizes this framing through a defined three-step sequence (positioning, dialogogic prompting, and critical integration) with an explicit risk–countermeasure governance map. The operational implementation of this framework, including a structured 32–1 post-session grid and de-identification protocols, is detailed in a companion brief report (Signorini and Paganin, 2026) (Table 1).

**Table 1:** Reflective risks and countermeasures of the SADAR method.

Risk	Description	Reflective countermeasures
Collusion/reduction of complexity	Embracing simplified, “clean” answers as clinical truth rather than as reflective stimuli.	Reiterate AI’s symbolic function; maintain a critical and dialectical stance; bring back to supervision.
Anthropomorphization	Attributing intentionality, authority, or empathic understanding to the AI system.	Use language emphasizing the metaphorical and symbolic nature of AI; avoid personification.
Delegitimization of clinical judgment	Treating AI as an “objective supervisor” or substituting its output for clinical reasoning.	Reaffirm individual professional responsibility; ensure priority always remains with human supervision.
Model biases and limitations (confabulation)	Outputs are influenced by training data and stylistic priors; they may be internally coherent but factually ungrounded.	Cultural contextualization; triangulation with theory and human supervision; systematic critical appraisal.
Linguistic suggestibility	Rhetorical prose may implicitly steer the therapist toward a pre-formed conclusion.	Treat AI as one voice among voices; slow down and interrogate proposals before accepting them.
Privacy, confidentiality and governance	Risk of data leakage, re-identification, and opaque log retention; potential non-compliance with regulatory frameworks (GDPR/HIPAA/APA/EFPA).	Avoid identifiable content; apply data minimization principles; ensure auditability; use compliant technical setups; adhere to institutional policies.

All SADAR uses are intended to align with the APA Ethics Code (American Psychological Association, 2017), EFPA Meta-Code (European Federation of Psychologists’ Associations, 2015), WHO digital health guidance (World Health Organization, 2019), and applicable data protection regimes (GDPR: European Union, 2016; HIPAA: U.S. Department of Health and Human Services, 2013; AI Act: European Parliament and Council of the European Union, 2024).

### Terminological and etymological note: dialogogic

“Dialogogic” is a purposeful neologism (δια-λόγος + ἀγωγή): A form of dialog that also conducts—that is, one that orients the relational field toward new meanings without directing or prescribing outcomes, symbolically guiding the internal voices without replacing human clinical judgment (Table 2).

**Table 2:** Dialogogic and agogic—concise definitions.

Term	Concise definition
Dialogic	Reciprocal, polyphonic exchange; co-construction of meaning.
Agogic	A conducting/formative vector that orients psychic/relational movement.
Dialogogic	A dialog that also conducts—that is, one that orients the relational field toward new meanings without directing or prescribing outcomes, symbolically guiding the internal voices without replacing human clinical judgment (Buber, 1958; Freire, 1970). In the SADAR, it is a structure designed to produce genuine discontinuity rather than confirmation.

### The SADAR method—scope of application, positioning, and exclusion

The SADAR (Authentic Relational Dialogogic Self-Exploratory System) does not propose AI as a subject or as a therapeutic protocol. AI is engaged as a Digital Analytic Third: A symbolically mediated presence that can evoke transferential and countertransferential movements, perturb consolidated arrangements, and catalyze new associations. Its scope of use is limited to the therapist’s internal reflection, individual or team supervision, and training. It is not indicated for direct interaction with the patient, for clinical decisions that substitute supervision, or for use in the absence of adequate governance and data protection safeguards.

Positioning with respect to CBT-oriented approaches and clinical chatbots: Unlike patient-facing tools that apply standardized protocols and aim at symptom reduction, the SADAR is positioned on the therapist’s side as a meta-therapeutic device. It opens symbolic space, preserves complexity, and avoids externalizing judgment, treating AI as one voice among voices to be critically weighed and re-appropriated in supervision.

Discouraged clinical conditions (non-exhaustive examples):

1. Acute crises / suicidality risk / imminent violence: Priority must be clinical and institutional management; individual use of the SADAR is not indicated.
2. Severe therapist disorganization/dissociation or a context lacking institutional holding: Risk of fragmentation.
3. Environments where confidentiality is not guaranteed or are non-compliant with GDPR/HIPAA standards: Risk of leakage or re-identification.
4. Absence of human supervision: Risk of delegitimizing clinical judgment.

#### Conceptual functioning (a three-step reflective process)

*Non-prescriptiveness note. The SADAR is neither a technique nor a protocol; it is a reflective framework that sustains an analytic posture, methodical doubt, and professional responsibility.*

Step 1—Therapist positioning (internal focusing). Delimit a specific internal difficulty (e.g., countertransferential node, stalemate, impasse, or internal voices in conflict).

The notion of “internal voices in conflict” invoked in Step 1 draws on a well-established theoretical lineage within psychoanalytic and group-analytic traditions. In the group-analytic framework developed by Foulkes (1964), the individual mind is constituted by an internalized group matrix whose multiple voices remain in ongoing dialog; pathology arises precisely when this internal polyphony is suppressed into pseudo-unitary self-narratives. Napolitani (1987), extending this tradition within gruppo-analisi antropoanalitica, further conceptualized the therapeutic encounter as a field in which both the therapist and patient bring their internal groups into relational play—a perspective that directly informs the SADAR’s use of AI as a symbolic perturbation capable of reactivating suppressed internal voices. This conception is also consistent with the Internal Family Systems model (Schwartz, 2001), which maps the psyche as a system of semi-autonomous parts requiring differentiation and dialog rather than suppression. Acknowledging this lineage positions Step 1 not as an idiosyncratic metaphor but as a clinically grounded practice with established theoretical precedents.

Guiding questions/examples: Which internal voices do I feel in tension (e.g., impatience ↔ care ↔ observation)? Which affect am I struggling to mentalize? What do I fear losing if I give up a reassuring yet reductive reading?

Step 2—Dialogogic prompting with AI (symbolic engagement). Address the model by assigning it an explicit symbolic function (e.g., “act as a Digital Analytic Third; list three tense readings of my countertransference; signal risks of collusion and bias”).

Examples of professional prompts (without identifying data): “Act as a Digital Analytic Third: name three alternative hypotheses about my impatience and ask me two critical questions.” “Depict three internal voices in conflict and propose a counter-scripted interpretation (pro/contra).”

The symbolic function assignment in Step 2 warrants further elaboration. Addressing an LLM as a ‘Digital Analytic Third’ is not a metaphorical flourish; it is a structurally functional instruction that reconfigures the model’s responses toward perturbation rather than confirmation. Research on LLM prompt sensitivity (Hwang et al., 2024; Kim et al., 2025) suggests that explicit role framing substantially shifts the type and diversity of outputs generated. In the SADAR, the prompt architecture requires three elements: A role instruction (symbolic function), a request for three alternative hypotheses, including one that challenges the therapist’s dominant reading, and an explicit invitation to identify the risk of collusion and bias in the therapist’s own framing. This three-part structure is designed to prevent the most common failure mode of AI-assisted reflection: a well-formed prompt that receives a well-formed confirmation. De-identification is non-negotiable: No patient-identifying data, direct quotes, names, or contextual details that could enable re-identification should ever appear in the prompt.

This failure mode—the well-formed prompt that receives a well-formed confirmation—is what we term gentle collusion (Signorini, 2025). The LLM produces exactly what the therapist’s implicit framing invites, not because the model errs, but because it succeeds at being responsive. The 3–2-1 structure of the SADAR is specifically designed as a counter-collusional device.

*Red flags of improper use: Excessive relief from “clean” simplifications, recourse to AI as an authority or decision shortcut, and reduction of subjective complexity.*

Step 3—Critical integration. It is the most demanding and clinically consequential phase of the SADAR process. Its function is not to select the ‘best’ AI hypothesis but to use the full range of AI outputs—including those the therapist finds implausible or disturbing—as perturbation material for clinical thinking. The integration process involves three sub-movements: Weighing (which output creates the most productive discomfort, and why?), contesting (actively arguing against each hypothesis and testing it against clinical evidence and theoretical consistency), and appropriating (reformulating the insight in the therapist’s own theoretical language before bringing it to human supervision). The output of Step 3 is not a conclusion but a set of questions—specifically, the hypotheses and risks identified in the 3–2-1 grid—to be brought to the next supervisory encounter. A therapist who leaves Step 3 with a settled answer has likely engaged in collusion rather than integration (Table 3).

**Table 3:** SADAR—operational steps (schematic).

Step	Essentials (What to do/examples)
① Positioning	Define a specific internal scenario (countertransference node, stalemate, internal conflict). Focus on affects and competing internal voices.
② Dialogogic prompting with AI	Assign an explicit symbolic function (“act as a Digital Analytic Third; list three tense readings; signal risks of collusion and bias”). Use only de-identified material.
③ Critical integration	Treat outputs as reflective stimuli, not prescriptions. Weigh and contest in human supervision; re-appropriate into personal clinical thinking.

### Clinical vignettes (illustrative, non-empirical)

Methodological note on vignettes. The clinical vignettes presented below are fictional and composite; they are not traceable to real cases and serve exclusively illustrative purposes. They demonstrate the method’s potential utility and limitations without constituting probative evidence of effectiveness. They do not shift the center of gravity of clinical judgment, which remains non-delegable and rests with the therapist and human supervision.

Vignette A—When the SADAR helps. A trainee therapist finishes a session marked by a long silence that felt unbearable. She writes in her Step 1 positioning: “I felt an urgent need to fill the silence—to say something useful, anything. I am worried I am too impatient for this patient.” She then prompts the AI: “Act as a Digital Analytic Third. Name three alternative hypotheses about my impatience and ask me two critical questions.” The AI returns three readings: Her impatience as a countertransferential signal mirroring the patient’s own frozen expectation, her impatience as a maternal concern about abandonment, and her impatience as a silent observational stance that she has not yet recognized as clinically useful. The third hypothesis stops her. She had not considered that her urge to speak might contain an observation she was refusing to trust. In Step 3, she contests the first hypothesis (too interpretive, too fast) and appropriates the third. She brings it to supervision not as a conclusion but as a question: “What if my silence is not a failure but a communication I have not yet decoded?” Countertransference becomes usable reflective material; analytic patience is restored.

Vignette B—When the SADAR misleads. An experienced therapist has been working for months with a patient who has survived severe relational trauma. After a particularly dense session, he uses the SADAR to try to disentangle what belongs to him and what belongs to the patient. In his positioning, he writes: “I feel a heaviness that I cannot locate—is it his grief or mine?” He prompts the AI for three hypotheses that differentiate his own resonances from the patient’s affective states. The AI responds with a clean taxonomy: Resonance A (therapist’s own unresolved loss), resonance B (introjected patient affect), and resonance C (relational field co-construction). The output is elegant and reassuring. He feels lighter. Something has been sorted. In Step 3, however, instead of contesting, he accepts—the taxonomy is too satisfying to argue with. He brings it to supervision as a finding rather than a question. His supervisor pauses: “But what if the heaviness is precisely what cannot be separated? What if the entanglement is the clinical material?” The AI’s clean separation had functioned as a defensive operation, confirming the therapist’s implicit wish to restore clear boundaries in a case that required tolerating their dissolution. Human supervision reintroduces complexity; the excessive relief from the “clean” output is retrospectively identified as the red flag it was.

## Ethics and risks (risk–countermeasure map)

### Discussion

The SADAR addresses a theoretical gap, integrating the psychoanalytic/group-analytic tradition (transference, countertransference, and internal multiplicity) with the use of AI, without flattening complexity into procedures or delegating authority to technology. We recognize the epistemic risks of LLMs—including confabulation, variable consistency, biases, and rhetorical persuasiveness—and propose practical mitigations: Treating AI as one voice among voices, prompting it with an explicitly stated symbolic function, triangulating outputs with theory and human supervision, and maintaining traceability of decisions. LLMs do not hallucinate in the perceptual sense of the term; rather, they confabulate—producing internally coherent linguistic constructs that lack factual grounding. This makes them particularly insidious in a reflective context, where narrative coherence already functions as a criterion of clinical plausibility. The contribution is conceptual and ethical [see also Signorini (2025)]: It offers reflective practices to explore transference, countertransference, and internal groupality without bypassing human responsibility.

The SADAR’s primary domain—the therapist’s internal reflection and clinical supervision—situates it within a well-established body of literature on reflective supervisory practice, which the present framework explicitly engages. Proctor (2001) identified three interrelated functions of clinical supervision—normative, formative, and restorative—each of which maps onto distinct dimensions of the SADAR process: Governance and ethical accountability (normative), the deepening of clinical understanding through hypothesis diversification (formative), and the containment of countertransferential activation between sessions (restorative). Hawkins and Shohet (2012) similarly emphasized that effective supervision requires a protected reflective space capable of tolerating complexity and ambiguity without premature closure—precisely the function that the SADAR’s three-step process is designed to sustain. Carroll (2009) further argued that reflective supervision must actively resist the pull toward confirmatory thinking, a risk that the SADAR addresses through its structured counter-hypothesis prompting and explicit collusion-detection mechanisms. More recently, Cioffi et al. (2025) provided preliminary empirical support for the feasibility of AI as a supervisory support tool, demonstrating that AI-generated feedback on clinical cases was rated by psychotherapy trainees as comparable to human supervisory input on technical and didactic dimensions, while still requiring human interpretive integration for relational depth—a finding that directly reinforces the SADAR’s positioning of AI as one voice among voices rather than as an autonomous supervisory authority.

Taken together, these theoretical and empirical convergences suggest that the SADAR addresses a structural vulnerability in AI-assisted clinical reflection that has received insufficient attention: The tendency of LLMs to produce outputs that are narratively satisfying and rhetorically coherent precisely when the therapist’s critical vigilance is most reduced—namely, immediately after a session. This phenomenon, which we term “*gentle collusion”* following Signorini (2025), is not a function of model error but of model success: The LLM generates exactly what the therapist’s implicit framing invites, thereby confirming rather than perturbing the dominant interpretation. The SADAR’s 3–2-1 structure is specifically designed as a counter-collusional device: The requirement to generate three hypotheses before prompting the AI, including at least one that contradicts the therapist’s preferred reading and introduces structural friction into the reflective process. This positions the SADAR not merely as a tool for self-reflection but as an institutional-level safeguard against the systematic biases that accompany post-session countertransferential processing.

### Limitations

**Figure 1:**
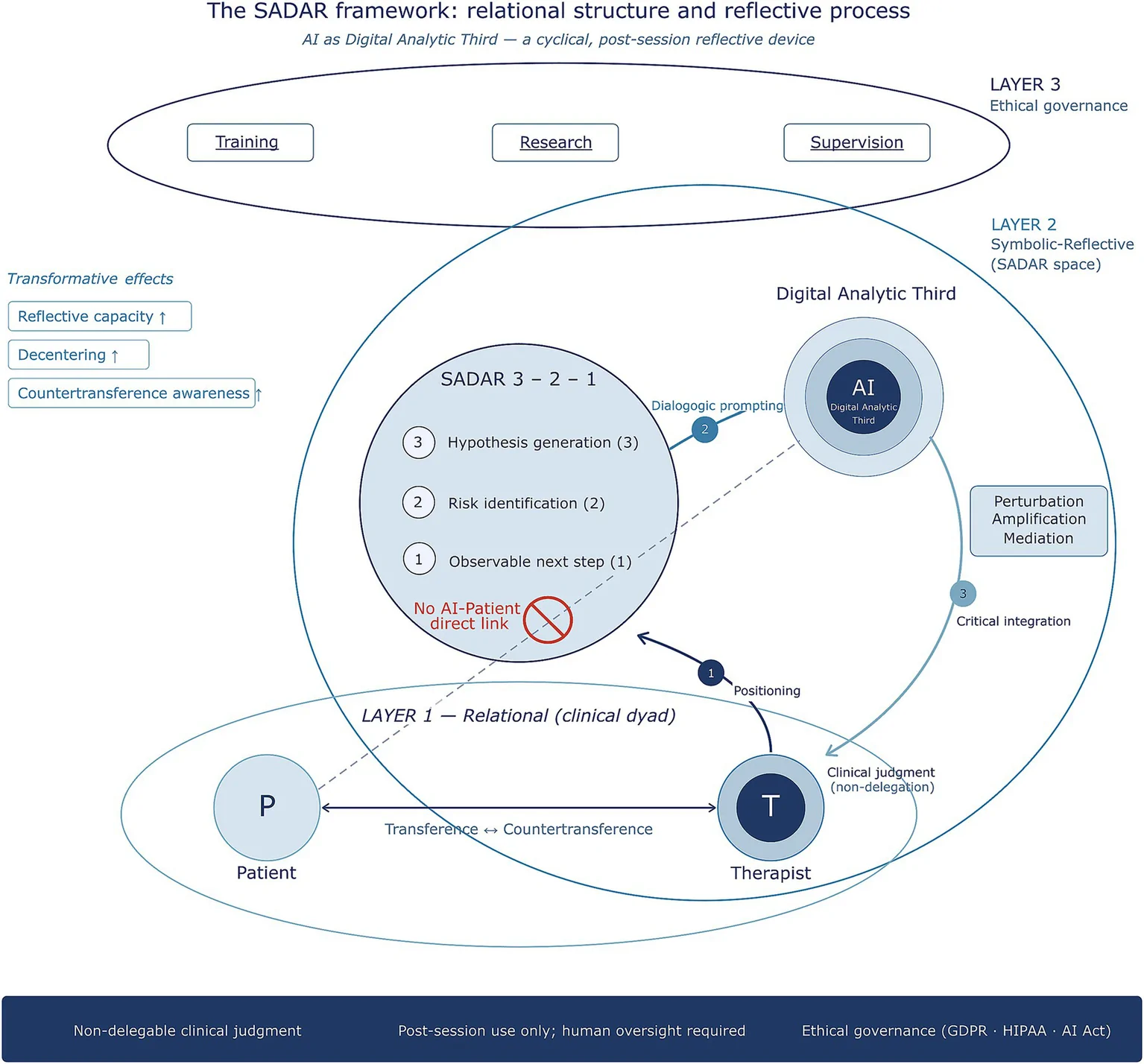
The SADAR framework: relational structure and dialogogic reflective process. AI as a digital analytic third—a cyclical, post-session reflective device. The framework is organized across three nested layers. Layer 1—relational (clinical dyad): The therapeutic relationship between patient (P) and therapist (T), governed by the transference ↔ countertransference dynamic; clinical judgment is non-delegable and remains exclusively with the therapist. Layer 2—symbolic-Reflective (SADAR space): the post-session reflective circuit, exclusive to the therapist–AI axis, structured as a three-step cyclical process: ① Positioning—the therapist delimits a specific countertransferential node or internal conflict; ② Dialogogic prompting—the therapist addresses AI by assigning it an explicit symbolic function (e.g., ‘act as a Digital Analytic Third; generate three alternative hypotheses; signal collusion risks’); and ③ Critical integration—the therapist weighs, contests, and re-appropriates AI outputs within clinical thinking and human supervision. The SADAR 3–2-1 structure (Hypothesis generation, risk identification, observable next step) operates within this layer. The AI exerts three transformative functions on the therapist’s reflective field: Perturbation, amplification, and mediation. The prohibition symbol marks the structural absence of any direct AI–Patient link. Left column: transformative effects produced over time (Reflective capacity ↑, Decentering ↑, Countertransference awareness ↑). Layer 3—ethical Governance: The encompassing frame, articulating three domains of application (training, research, and supervision) and three non-negotiable principles: non-delegable clinical judgment, post-session use only with required human oversight, and ethical governance (GDPR, HIPAA, and AI Act compliance). The figure represents a post-session reflective device and does not imply real-time AI presence during the clinical session.

This is a conceptual article supported by illustrative vignettes and a qualitative risk map; it does not include empirical data. Effects are likely context-dependent and moderated by therapist competence, setting, and governance. LLM confabulation and consistency issues persist and must be mitigated through the practices outlined above. As confabulated outputs are narratively coherent, they may be especially difficult to identify as clinically unreliable in the post-session reflective state, where cognitive vigilance is reduced (Figure 1).

Several additional limitations warrant explicit acknowledgment. First, although the SADAR is grounded primarily in psychoanalytic, group-analytic, and IFS theoretical traditions, its transferability to other clinical orientations—such as cognitive–behavioral, systemic, or humanistic approaches—has not been examined. The constructs of countertransference, internal multiplicity, and the symbolic third may carry different theoretical weight and operational meanings across orientations, and adaptation of the framework to non-psychodynamic settings would require systematic conceptual work beyond the scope of this article.

Second, the SADAR is described at a level of abstraction that is intentionally model-agnostic; however, its practical implementation is necessarily conditioned by the specific LLM used, the platform’s data retention policies, the version of the model, and the quality of de-identification applied to the prompt. Variation in these parameters may substantially affect both the quality of AI outputs and the privacy risk profile. The framework assumes a technically and ethically adequate implementation context that may not be universally available, particularly in low-resource clinical settings or jurisdictions with less developed digital governance infrastructure.

Third, the risk of method drift deserves attention. The SADAR’s counter-collusional function depends on structured adherence to the 3–2-1 format and on the therapist’s willingness to genuinely entertain disconfirming hypotheses. In practice, therapists may abbreviate the protocol, skip the counter-hypothesis step, or unconsciously frame prompts in ways that reinvite confirmation. The absence of an external supervisor in the post-session reflective moment—by design—means that method fidelity cannot be independently verified without dedicated audit mechanisms, such as prompt log review. Future implementations should incorporate fidelity monitoring as a standard component of governance (Table 4).

**Table 4:** Open evaluation questions and testable hypotheses.

1. Therapist’s reflective capacity. Hypothesis: The SADAR increases tolerance of uncertainty, countertransferential awareness, and interpretive flexibility. Measures: Reflective Functioning Questionnaire (RFQ; Fonagy et al., 2016); qualitative pre–post analysis coded for hypothesis diversity. 2. Supervision and teamwork. Hypothesis: The SADAR improves case discussion quality, hypothesis plurality, and prevention of collusion. Measures: Therapist Response Questionnaire (Zittel Conklin and Westen, 2005); Post-Session Therapist Questionnaire (Negri et al., 2022). 3. Safety, ethics, and governance. Hypothesis: Structured de-identification protocols reduce privacy violation risk. See companion report (Signorini and Paganin, 2026). 4. Moderators and mediators. Hypothesis: Effects are mediated by reflective style and tolerance of ambiguity and moderated by therapist competence, setting, and institutional culture. 5. Therapist drift. Hypothesis: The SADAR reduces longitudinal drift toward self-consistent formulations (Waller, 2009), operationalized via semantic variance analysis of 3–2-1 grids; design: N-of-1 with blind hypothesis coding.

### Future directions (questions and hypotheses)

To facilitate future empirical investigation of the hypotheses outlined above, key SADAR constructs can be operationalized as follows. Reflective capacity may be assessed using the Reflective Functioning Questionnaire (RFQ; Fonagy et al., 2016), which provides a validated self-report measure of mentalizing capacity, or through qualitative pre–post analysis of therapists’ case formulation narratives coded for hypothesis diversity and tolerance of ambiguity. Countertransferential awareness can be indexed via the Therapist Response Questionnaire (Zittel Conklin and Westen, 2005), administered post-session, complemented by the Post-Session Therapist Questionnaire (Negri et al., 2022), which specifically captures elaborative reflexivity in the inter-session period—the temporal window in which the SADAR is designed to operate. Hypothesis diversity—the core process indicator of the method—can be operationalized as the number of distinct theoretical domains represented across the three alternative hypotheses generated per session (e.g., relational, intrapsychic, and contextual), assessed by independent raters blind to condition. Completion time and frequency of use provide additional feasibility indicators that are accessible without specialist measurement infrastructure. Governance compliance can be audited through prompt log review against pre-specified de-identification criteria, offering a traceable process measure aligned with GDPR and AI Act requirements (Appendix Table A1).

## Conclusion

More broadly, the SADAR contributes a theoretical model for how AI can be incorporated into clinical practice without eroding the epistemic and relational conditions that make psychotherapy effective. The central argument is not that AI enhances therapist reflection, but that structured dialogogic engagement with AI—subject to explicit governance, symbolic framing, and mandatory human supervision—can expand the reflective field in ways that are both clinically meaningful and empirically testable. As the integration of AI into mental health practice accelerates, frameworks that preserve rather than displace clinical complexity will become increasingly necessary. The SADAR is offered as one such framework—Provisional, revisable, and grounded in the conviction that the most important question is not what AI can do for therapy, but what kind of thinking it invites in the therapist.

The SADAR proposes AI as a reflective-symbolic co-presence capable of expanding the therapist’s reflective field and deepening countertransferential awareness, without replacing supervision. It does not prescribe techniques but reactivates dialogic, agogic, ethical, and imaginative functions at the interface between psychoanalysis and technology. Its adoption requires explicit and verifiable human accountability and governance. All outputs should remain within reflective supervision protocols and must never replace or supplement direct patient data.

## Data Availability

The original contributions presented in the study are included in the article/supplementary material, further inquiries can be directed to the corresponding author/s.

## Appendix Prompt Templates (for Clinician Use)

**Table A1:** Prompt templates for clinician use.

*Usage note: Never include patient-identifying data. Treat all AI outputs as reflective stimuli only; integrate within human supervision. All uses must comply with GDPR/HIPAA and applicable data protection regulations.*
Prompt 1—Reflective countertransference check (de-identified):
*“Act as a Digital Analytic Third. List three alternative hypotheses about my reaction (impatience vs. care vs. observation); pose two critical questions that challenge my preferred reading; signal one possible bias/collusion risk.”*
Prompt 2—Bias/collusion safeguard (de-identified):
*“As a Digital Analytic Third, generate: (a) three tensions I might be downplaying; (b) one ‘clean but reductive’ interpretation to avoid; (c) a supervision-oriented step that keeps complexity and responsibility with me.”*
